# What are the influencing factors for chronic pain following TAPP inguinal hernia repair: an analysis of 20,004 patients from the Herniamed Registry

**DOI:** 10.1007/s00464-017-5893-2

**Published:** 2017-10-26

**Authors:** H. Niebuhr, F. Wegner, M. Hukauf, M. Lechner, R. Fortelny, R. Bittner, C. Schug-Pass, F. Köckerling

**Affiliations:** 1 Hanse-Hernia Center, Alte Holstenstrasse 16, 21031 Hamburg, Germany; 2 StatConsult GmbH, Halberstädter Strasse 40 a, 39112 Magdeburg, Germany; 3 Department of Surgery, Paracelsus Medical University, Müllner Hauptstrasse 48, 5020 Salzburg, Austria; 4 Department of General, Visceral and Oncologic Surgery, Wilhelminen Hospital, Montleartstrasse 37, 1160 Vienna, Austria; 5 Winghofer Medicum, Hernia Center, Winghofer Strasse 42, 72108 Rottenburg am Neckar, Germany; 6 Department of Surgery and Center for Minimally Invasive Surgery, Academic Teaching Hospital of Charité Medical School, Vivantes Hospital, Neue Bergstrasse 6, 13585 Berlin, Germany

**Keywords:** Inguinal hernia, TAPP, Chronic pain, Complications, Hernia registry

## Abstract

**Background:**

In inguinal hernia repair, chronic pain must be expected in 10–12% of cases. Around one-quarter of patients (2–4%) experience severe pain requiring treatment. The risk factors for chronic pain reported in the literature include young age, female gender, perioperative pain, postoperative pain, recurrent hernia, open hernia repair, perioperative complications, and penetrating mesh fixation. This present analysis of data from the Herniamed Hernia Registry now investigates the influencing factors for chronic pain in male patients after primary, unilateral inguinal hernia repair in TAPP technique.

**Methods:**

In total, 20,004 patients from the Herniamed Hernia Registry were included in uni- and multivariable analyses. For all patients, 1-year follow-up data were available.

**Results:**

Multivariable analysis revealed that onset of pain at rest, on exertion, and requiring treatment was highly significantly influenced, in each case, by younger age (*p* < 0.001), preoperative pain (*p* < 0.001), smaller hernia defect (*p* < 0.001), and higher BMI (*p* < 0.001). Other influencing factors were postoperative complications (pain at rest *p* = 0.004 and pain on exertion *p* = 0.023) and penetrating compared with glue mesh fixation techniques (pain on exertion *p* = 0.037).

**Conclusions:**

The indication for inguinal hernia surgery should be very carefully considered in a young patient with a small hernia and preoperative pain.

After mesh-based inguinal hernia repair 10–12% of patients experience at least a level of moderate pain that impacts daily activities [1–6]. Chronic pain is defined as any pain reported by the patient at or beyond 3 months postoperatively [2]. More than one-quarter of these patients (2–4%) have moderate to severe pain [2, 5, 6]. Risk factors for chronic postoperative inguinal pain include young age, female gender, high preoperative pain, early high postoperative pain, recurrent hernia, and open hernia repair [1–6].

In all statements in the guidelines of the international hernia societies, laparo-endoscopic techniques are associated with less chronic pain than the Lichtenstein repair [7–11]. However, after laparo-endoscopic inguinal hernia repair, 2–5% of patients may still suffer from persistent pain influencing everyday activities, and about 0.4% are referred to pain clinics [12].

On the basis of three meta-analyses of randomized controlled trials [13–15] mesh fixation in total extraperitoneal patch plasty (TEP) can only be recommended in large medial/direct (EHS MIII) hernias [10].

In the Guidelines of the International Endohernia Society, a recommendation is given for consideration of non-fixation of the mesh in transabdominal preperitoneal patch plasty (TAPP) inguinal hernia repair in types LI, II and MI, II (EHS classification) [9, 10]. For TAPP repair of larger defects (LIII, MIII), the mesh should be fixed [9, 10]. In TAPP inguinal hernia repair, mesh fixation is still used in 66.1% of all primary unilateral cases in men [16].

Considering the fact that five meta-analyses [17–21] compared non-penetrating vs. mechanical mesh fixation, high-quality evidence could be expected. However, the meta-analyses can only conclude that the evidence is mostly of low or moderate quality, or that more high-quality multicenter studies are needed [22]. In view of the guidelines, fibrin glue should be considered for fixation to minimize the risk of postoperative acute and chronic pain [9, 10].

In a nationwide registry-based study, no differences were found in the frequency of recurrence and chronic pain between permanent and no/non-permanent fixation of the mesh in endoscopic inguinal hernia repair [23].

Another registry-based study from the Danish Hernia Database also found no difference in chronic pain after mesh fixation with fibrin glue vs. tacks in TAPP inguinal hernia repair [24].

The following analysis of data from the Herniamed Registry now investigates the influencing factors for chronic pain in male patients after primary, unilateral inguinal hernia repair in TAPP technique.

## Methods

As of October 10, 2016, 577 participating hospitals and office-based surgeons mainly from Germany, Austria, and Switzerland had entered prospective data into the internet-based Herniamed Hernia Registry on their patients who had undergone routine hernia surgery and signed an informed consent agreeing to participate [25]. As part of the information provided to patients regarding participation in the Herniamed Quality Assurance Study and signing the informed consent declaration, all patients are informed that the treating hospital or medical practice would like to be informed about any problems occurring after the operation and that the patient has the opportunity to attend for clinical examination.

This present study analyzed the prospective data collected for all male patients who had been operated on with an endoscopic TAPP technique for repair of a primary unilateral inguinal hernia in the period September 01, 2009, up to and including September 01, 2015. On 1-year follow-up, the general practitioners and patients were asked through questionnaire about any pain at rest, pain on exertion, and chronic pain requiring treatment. If chronic pain is reported by the general practitioner or patient, patients can be requested to attend for clinical examination. A recent publication has provided impressive evidence of the role of patient-reported outcomes for the identification of chronic pain rates after groin hernia repair [26]. Only those patients for whom 1-year follow-up results were available were included in the analysis. Other inclusion criteria included age ≥ 16 years and only medial/lateral/combined types of inguinal hernia based on the EHS classification [27].

**Fig. 1:**
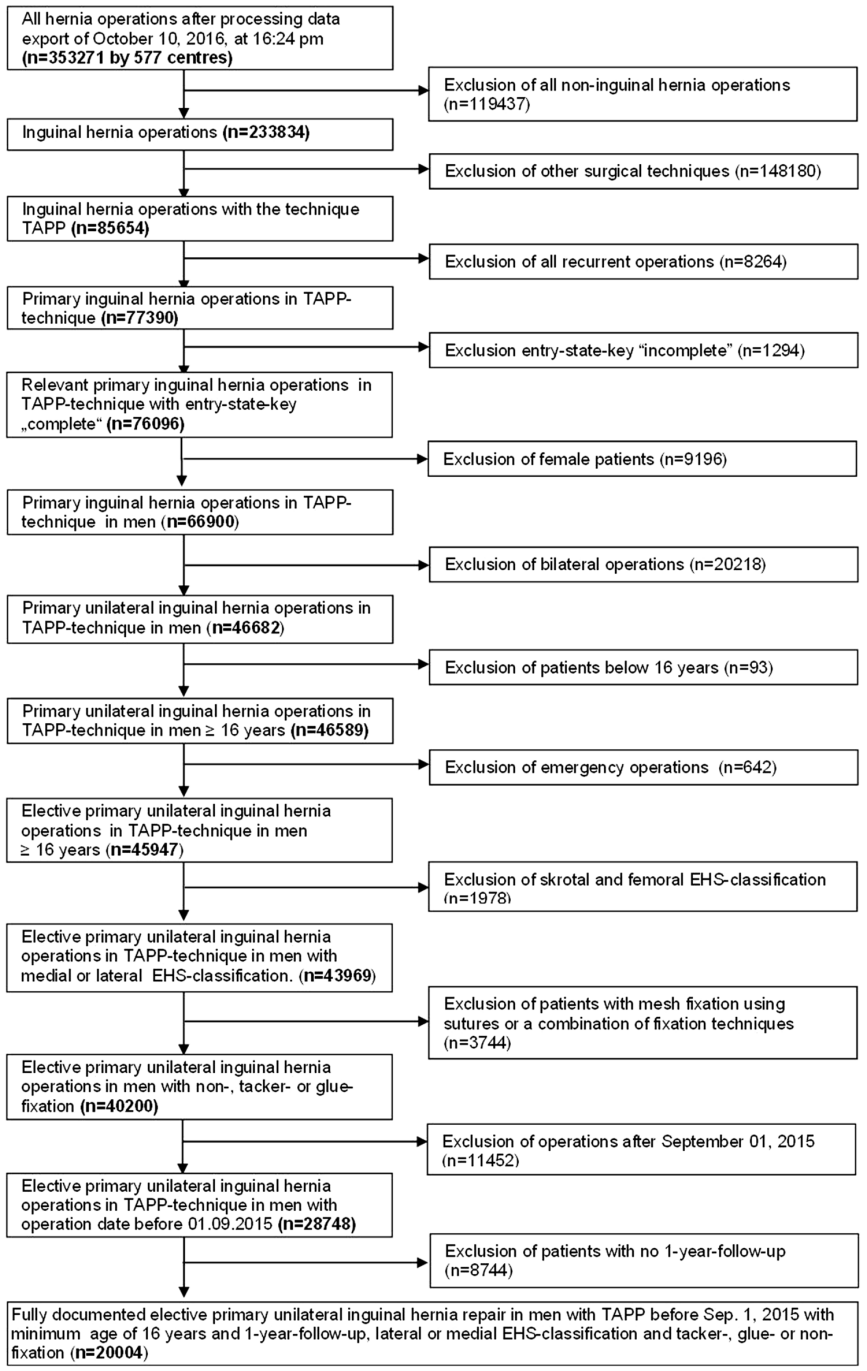
Flowchart of patient inclusion

In total, 20,004 were included in uni- and multivariable analyses for investigation of the influencing factors for the development of chronic pain following TAPP inguinal hernia repair (Fig. 1).

All analyses were performed with the software SAS 9.4 (SAS Institute Inc. Cary, NC, USA) and intentionally calculated to a full significance level of 5%, i.e., they were not corrected in respect of multiple tests, and each *p* value ≤ 0.05 represents a significant result.

To first discern differences between the groups in unadjusted analyses, Fisher’s exact test was used for categorical outcome variables, and the robust *t* test (Satterthwaite) for continuous variables. For mesh size (cm^2^), a logarithmic transformation was applied and the re-transformed mean and range of dispersion are given.

To identify influence factors in multivariable analyses, binary logistic regression models for pain at rest, pain on exertion, and chronic pain requiring treatment were used. Potential influence factors were ASA score (I/II/III/IV), age (years), BMI (kg/m^2^), mesh size (cm^2^), defect size (I/II/III), risk factors (yes/no), preoperative pain (yes/no/unknown), EHS classification (lateral/medial/combined), postoperative complication (yes/no), and mesh fixation (no fixation/tacker/glue). Estimates for odds ratio (OR) and the corresponding 95% confidence interval based on the Wald test were given. For influence variables with more than two categories, pairwise odds ratios were given. For age (years) the 10-year OR estimate, for BMI (kg/m^2^) the five-point OR estimate, and for mesh size (cm^2^) the 10-point OR estimate were given. The results are presented in tabular form, sorted by descending impact.

## Results

In total, 20,004 patients were included in the analysis exploring the influence exerted by the fixation technique as well as by other influencing variables on the rate of pain at rest, pain on exertion, and chronic pain requiring treatment (Fig. 1). Of these, 8799 patients had no fixation (44.0%), 6387 (31.9%) only tacker fixation, and 4818 patients (24.1%) only glue fixation.

The patient group in whom the mesh was fixed with a tacker was on average the oldest and had also the highest BMI (Table 1). While there were significant differences in age and BMI between the two groups due to the large patient number, these were not clinically relevant. The meshes in the patient group with no fixation were smaller (Table 1). Besides, meshes with glue fixation had the fewest (8.5%) and meshes with tacker fixation the most (14.1%) ASA III/IV patients (Table 2). The operations with no fixation were most commonly encountered for small hernia defect sizes (17.4%). As regards the defect localization, for lateral EHS classification no or only glue fixation was mainly used, whereas for medial EHS classification tacker fixation was most common (Table 2). Preoperative pain was less common among patients with tacker mesh fixation (59.4%) than in patients with glue mesh fixation (67.3%) or no mesh fixation (66.3%). Drain placement was most commonly used for patients with no mesh fixation (8.4%). In terms of the risk factors, mesh fixation with tackers or glue was more common in patients who continued to take platelet aggregation inhibitors (6.2 vs. 7.8% and 7.6%).

**Table 1:** Mean age, BMI, and mesh size in male patients with primary unilateral inguinal hernia repair in TAPP technique

		Non-fixation	Tacker	Glue	*p*
Age (years)	Median ± STD	55.0 ± 15.6	58.8 ± 14.7	56.4 ± 15.0	< 0.001
BMI	Mean ± STD	25.9 ± 3.3	26.0 ± 3.4	25.8 ± 3.4	< 0.001
Mesh size (cm^2^)	MW	146.3 [145.2; 147.5]	149.9 [148.7; 151.1]	151.1 [150.1; 152.2]	< 0.001

**Table 2:** Patient and operative characteristics in relation to mesh fixation and unadjusted tests for significant differences

	Non-fixation		Tacker		Glue		*p*
	*n*	%	*n*	%	*n*	%	
ASA score							
I	3043	34.58	1864	29.18	1946	40.39	< 0.001
II	4737	53.84	3621	56.69	2461	51.08	
III/IV	1019	11.58	902	14.12	411	8.53	
Defect size							
I (< 1.5 cm)	1533	17.42	727	11.38	683	14.18	< 0.001
II (1.5–3 cm)	6072	69.01	3939	61.67	3200	66.42	
III (> 3 cm)	1194	13.57	1721	26.95	935	19.41	
EHS classification							
Combined	1128	12.82	633	9.91	394	8.18	< 0.001
Lateral	5483	62.31	3718	58.21	3142	65.21	
Medial	2188	24.87	2036	31.88	1282	26.61	
Drainage							
Yes	736	8.36	346	5.42	148	3.07	< 0.001
No	8063	91.64	6041	94.58	4670	96.93	
Risk factors							
Total							
Yes	2248	25.55	1665	26.07	1233	25.59	0.747
No	6551	74.45	4722	73.93	3585	74.41	
COPD							
Yes	368	4.18	313	4.90	195	4.05	0.044
No	8431	95.82	6074	95.10	4623	95.95	
Diabetes							
Yes	353	4.01	302	4.73	207	4.30	0.100
No	8446	95.99	6085	95.27	4611	95.70	
Aortic aneurysm							
Yes	27	0.31	30	0.47	12	0.25	0.103
No	8772	99.69	6357	99.53	4806	99.75	
Immunosuppression							
Yes	47	0.53	34	0.53	15	0.31	0.151
No	8752	99.47	6353	99.47	4803	99.69	
Corticoid							
Yes	58	0.66	39	0.61	43	0.89	0.173
No	8741	99.34	6348	99.39	4775	99.11	
Smoking							
Yes	1034	11.75	659	10.32	540	11.21	0.021
No	7765	88.25	5728	89.68	4278	88.79	
Coagulopathy							
Yes	117	1.33	64	1.00	34	0.71	0.003
No	8682	98.67	6323	99.00	4784	99.29	
Antiplatelet medication							
Yes	544	6.18	499	7.81	365	7.58	< 0.001
No	8255	93.82	5888	92.19	4453	92.42	
Anticoagulation therapy							
Yes	133	1.51	118	1.85	68	1.41	0.134
No	8666	98.49	6269	98.15	4750	98.59	
Preoperative pain							
Yes	5829	66.25	3796	59.43	3241	67.27	< 0.001
No	2515	28.58	2069	32.39	1210	25.11	
Unknown	455	5.17	522	8.17	367	7.62	

Unadjusted analysis of the relationship between the fixation technique and the intra- and postoperative complications, recurrence rate as well as pain at rest, on exertion, and requiring treatment on 1-year follow-up is given in detail in Table 3. For postoperative complications, pain on exertion and pain requiring treatment differences are identified in relation to the fixation technique used. For postoperative complication these are largely due to an increased seroma rate (no fixation 0.7% vs. tacker fixation 2.1% vs. glue fixation 3.9%). For operations with no mesh fixation, the rate of pain on exertion (no fixation 10.1% vs. tacker fixation 9.4% vs. glue fixation 8.8%) and pain requiring treatment (no fixation 3.0% vs. tacker fixation 2.4% vs. glue fixation 2.3%) was somewhat higher than in the groups with tacker or glue mesh fixation.

**Table 3:** Outcome variables in relation to mesh fixation and unadjusted tests for significant differences

	Non-fixation		Tacker		Glue		*p*
	*n*	%	*n*	%	*n*	%	
Intraoperative complication							
Total							
Yes	71	0.81	67	1.05	55	1.14	0.114
No	8728	99.19	6320	98.95	4763	98.86	
Bleeding							
Yes	48	0.55	52	0.81	35	0.73	0.120
No	8751	99.45	6335	99.19	4783	99.27	
Injury							
Total							
Yes	42	0.48	31	0.49	27	0.56	0.790
No	8757	99.52	6356	99.51	4791	99.44	
Vascular							
Yes	23	0.26	17	0.27	11	0.23	0.914
No	8776	99.74	6370	99.73	4807	99.77	
Bowel							
Yes	6	0.07	4	0.06	4	0.08	0.918
No	8793	99.93	6383	99.94	4814	99.92	
Bladder							
Yes	4	0.05	6	0.09	5	0.10	0.394
No	8795	99.95	6381	99.91	4813	99.90	
Nerve							
No	8799	100.0	6387	100.0	4818	100.0	1.000
Postoperative complications							
Total							
Yes	159	1.81	192	3.01	231	4.79	< 0.001
No	8640	98.19	6195	96.99	4587	95.21	
Bleeding							
Yes	76	0.86	50	0.78	34	0.71	0.602
No	8723	99.14	6337	99.22	4784	99.29	
Seroma							
Yes	61	0.69	133	2.08	189	3.92	< 0.001
No	8738	99.31	6254	97.92	4629	96.08	
Infection							
Yes	6	0.07	7	0.11	2	0.04	0.407
No	8793	99.93	6380	99.89	4816	99.96	
Bowel							
Yes	9	0.10	1	0.02	1	0.02	0.041
No	8790	99.90	6386	99.98	4817	99.98	
Wound healing disorders							
Yes	7	0.08	5	0.08	8	0.17	0.250
No	8792	99.92	6382	99.92	4810	99.83	
Ileus							
Yes	8	0.09	2	0.03	3	0.06	0.362
No	8791	99.91	6385	99.97	4815	99.94	
Complication-related reoperations							
Yes	89	1.01	55	0.86	35	0.73	0.226
No	8710	98.99	6332	99.14	4783	99.27	
Recurrence on 1-year follow-up							
Yes	91	1.03	56	0.88	41	0.85	0.467
No	8708	98.97	6331	99.12	4777	99.15	
Pain at rest on 1-year follow-up							
Yes	466	5.30	309	4.84	225	4.67	0.214
No	8333	94.70	6078	95.16	4593	95.33	
Pain on exertion on 1-year follow-up							
Yes	884	10.05	599	9.38	422	8.76	0.045
No	7915	89.95	5788	90.62	4396	91.24	
Pain requiring treatment on 1-year follow-up							
Yes	260	2.95	152	2.38	109	2.26	0.021
No	8539	97.05	6235	97.62	4709	97.74	

Tables 2 and 3 show, in some cases, significant differences in the influencing factors and thus also in outcomes in relation to fixation vs. non-fixation. Accordingly, the unadjusted analysis results permit only limited comparability and therefore call for multivariable analysis.

## Multivariable analysis

### Pain at rest on 1-year follow-up

The results of analysis of pain at rest on 1-year follow-up are summarized in Table 4 (model fitting: *p* < 0.001). Pain at rest was highly significantly influenced by the presence of preoperative pain, by age, BMI, and the hernia defect size (in each case *p* < 0.001). In higher age (10-year OR 0.880 [0.839; 0.924]; *p* < 0.001) the risk of pain at rest was lower, whereas for higher BMI (five-point OR 1.225 [1.124; 1.334]; *p* < 0.001), presence of preoperative pain (yes vs. no: OR 1.862 [1.574; 2.201]; *p* < 0.001), and smaller hernia defect (I vs. III: OR 1.619 [1.298; 2.021]; *p* < 0.001) it was higher. Additionally, postoperative complications led also to a higher risk of onset of pain at rest (OR 1.613 [1.162; 2.239]; *p* = 0.004). There was no evidence of the fixation technique having any influence on the risk of onset of pain at rest.

**Table 4:** Multivariable analysis of influencing factors for pain at rest on 1-year follow-up

Parameter	*p* Value	Category	*p* Value paired	OR estimate	95% CI	
Preoperative pain	< 0.001	Yes vs. no	< 0.001	1.862	1.574	2.201
		Yes vs. unknown	0.149	1.214	0.933	1.581
		No vs. unknown	0.004	0.652	0.486	0.875
Age (10-year OR)	< 0.001			0.880	0.839	0.924
BMI (5-point OR)	< 0.001			1.225	1.124	1.334
Defect size	< 0.001	I (< 1.5 cm) vs. II (1.5–3 cm)	< 0.001	1.453	1.233	1.714
		I (< 1.5 cm) vs. III (> 3 cm)	< 0.001	1.619	1.298	2.021
		II (1.5–3 cm) vs. III (> 3 cm)	0.244	1.114	0.929	1.336
Postoperative complication	0.004	Yes vs. no		1.613	1.162	2.239
Fixation	0.354	Glue vs. tacks	0.184	0.886	0.741	1.059
		Glue vs. non-fixation	0.210	0.899	0.761	1.062
		Tacks vs. non-fixation	0.850	1.015	0.872	1.181
Risk factors	0.511	Yes vs. no		1.053	0.903	1.229
ASA score	0.513	I vs. II	0.248	0.913	0.783	1.065
		I vs. III/IV	0.552	0.923	0.710	1.201
		II vs. III/IV	0.924	1.011	0.806	1.269
Mesh size (10-point OR)	0.652			0.992	0.959	1.026
EHS classification	0.745	Combined vs. lateral	0.609	1.057	0.856	1.305
		Combined vs. medial	0.449	1.094	0.867	1.379
		Lateral vs. medial	0.649	1.035	0.892	1.201

### Pain on exertion on 1-year follow-up

Pain on exertion on 1-year follow-up, whose analysis results are summarized in Table 5 (model fitting: *p* < 0.001), was significantly influenced by age, preoperative pain, hernia defect size, BMI (in each case *p* < 0.001), mesh size (*p* = 0.031), postoperative complications (*p* = 0.023), and the fixation technique (*p* = 0.037). A higher age (10-year OR 0.796 [0.768; 0.825]; *p* < 0.001) led to a lower risk and preoperative pain (yes vs. no: OR 1.516 [1.349; 1.705]; *p* < 0.001) to a higher risk of onset of pain on exertion. Small defect sizes (I vs. III: OR 1.605 [1.354; 1.902]; *p* < 0.001), higher BMI (five-point OR 1.180 [1.104; 1.260]; *p* < 0.001), and onset of postoperative complications (yes vs. no: OR 1.364 [1.045; 1.780]; *p* = 0.023) increased the risk of pain on exertion.

**Table 5:** Multivariable analysis of influencing factors for pain on exertion on 1-year follow-up

Parameter	*p* Value	Category	*p* Value paired	OR estimate	95% CI	
Age (10-year OR)	< 0.001			0.796	0.768	0.825
Preoperative pain	< 0.001	Yes vs. no	< 0.001	1.516	1.349	1.705
		Yes vs. unknown	0.040	1.236	1.010	1.513
		No vs. unknown	0.067	0.815	0.655	1.015
Defect size	< 0.001	I (< 1.5 cm) vs. II (1.5–3 cm)	< 0.001	1.317	1.163	1.492
		I (< 1.5 cm) vs. III (> 3 cm)	< 0.001	1.605	1.354	1.902
		II (1.5–3 cm) vs. III (> 3 cm)	0.006	1.218	1.060	1.401
BMI (5-point OR)	< 0.001			1.180	1.104	1.260
Postoperative complication	0.023	Yes vs. no		1.364	1.045	1.780
Mesh size (10-point OR)	0.031			0.971	0.946	0.997
Fixation	0.037	Glue vs. tacks	0.010	0.839	0.734	0.959
		Glue vs. non-fixation	0.123	0.906	0.800	1.027
		Tacks vs. non-fixation	0.183	1.080	0.964	1.209
ASA score	0.088	I vs. II	0.031	0.882	0.787	0.988
		I vs. III/IV	0.496	0.932	0.760	1.142
		II vs. III/IV	0.548	1.056	0.883	1.263
Risk factors	0.416	Yes vs. no		1.049	0.934	1.179
EHS classification	0.518	Combined vs. lateral	0.727	1.029	0.875	1.210
		Combined vs. medial	0.701	0.966	0.811	1.151
		Lateral vs. medial	0.254	0.939	0.842	1.046

The use of a larger mesh reduced the risk of pain on exertion (10-point OR 0.971 [0.946; 0.997]; *p* = 0.031). There was also evidence of the influence of the fixation technique, (*p* = 0.037), revealing that tacker compared with glue fixation led to a higher rate of pain on exertion (OR 1.192 [1.043; 1.362]; *p* = 0.010).

### Chronic pain requiring treatment on 1-year follow-up

The results of analysis of the influencing factors for pain requiring treatment are shown in Table 6 (model fitting: *p* < 0.001). Here, too, the risk of onset of chronic pain requiring treatment was highly significantly affected by the hernia defect size, age, BMI, and preoperative pain (in each case *p* < 0.001). The rate of chronic pain requiring treatment was, in particular, negatively influenced by small defect sizes (I vs. III: OR 1.996 [1.482; 2.688]; *p* < 0.001), higher BMI (five-point OR 1.319 [1.181; 1.473]; *p* < 0.001) as well as by preoperative pain (yes vs. no: OR 1.819 [1.441; 2.296]; *p* < 0.001). On the other hand, higher age (10-year OR 0.842 [0.788; 0.899]; *p* < 0.001) resulted in a lower risk of chronic pain requiring treatment.

**Table 6:** Multivariable analysis of influencing factors for chronic pain requiring treatment

Parameter	*p* Value	Category	*p* Value paired	OR estimate	95% CI	
Defect size	< 0.001	I (< 1.5 cm) vs. II (1.5–3 cm)	< 0.001	1.853	1.500	2.289
		I (< 1.5 cm) vs. III (> 3 cm)	< 0.001	1.996	1.482	2.688
		II (1.5–3 cm) vs. III (> 3 cm)	0.572	1.077	0.832	1.394
Age (10-year OR)	< 0.001			0.842	0.788	0.899
BMI (5-point OR)	< 0.001			1.319	1.181	1.473
Preoperative pain	< 0.001	Yes vs. no	< 0.001	1.819	1.441	2.296
		Yes vs. unknown	0.794	1.048	0.739	1.486
		No vs. unknown	0.006	0.576	0.388	0.854
Risk factors	0.079	Yes vs. no		1.203	0.979	1.478
Mesh size (10-point OR)	0.167			0.966	0.921	1.014
Fixation	0.202	Glue vs. tacks	0.280	0.870	0.675	1.120
		Glue vs. non-fixation	0.074	0.810	0.642	1.021
		Tacks vs. non-fixation	0.501	0.931	0.755	1.147
ASA score	0.462	I vs. II	0.405	0.914	0.740	1.129
		I vs. III/IV	0.223	0.803	0.565	1.142
		II vs. III/IV	0.403	0.879	0.649	1.189
EHS classification	0.591	Combined vs. lateral	0.618	1.076	0.807	1.436
		Combined vs. medial	0.341	1.167	0.849	1.605
		Lateral vs. medial	0.436	1.085	0.884	1.331
Postoperative complication	0.729	Yes vs. no		0.902	0.504	1.615

### Additional analysis

An additional analysis was included to show, not only qualitatively but also quantitatively, the results for the impact of age and BMI on chronic pain. This revealed that age ≤ 40 years was associated with the highest rates of pain at rest (6.4%), pain on exertion (13.7%), and pain requiring treatment (3.6%) on 1-year follow-up. Patients between > 40 and 60 years had mean pain rates (5.5, 11.6, and 3.1%, respectively) and patients > 60 years had the lowest pain rates (3.9, 6.0, and 1.8%, respectively). Patients with BMI of 18.5–24.9 (WHO classification: normal weight) had the lowest pain rates (4.2, 8.5, and 2.1%, respectively), those with BMI between 25.0 and 29.0 (WHO classification: overweight) had average rates (5.5, 10.0, and 2.7%, respectively) and those with BMI ≥ 30 (WHO classification: obesity) the highest pain rates (6.3, 11.5 , and 4.1%, respectively).

## Discussion

The present analysis of data from the Herniamed Hernia Registry for 20,004 male patients with elective primary, unilateral inguinal hernia repair in TAPP technique and with 1-year follow-up results has once again confirmed that, as reported in the literature, a pain at rest rate of 4–5%, pain on exertion of 8–10%, and pain requiring treatment of 2–3% must be expected [1–6]. In this selected patient group with laparoscopic inguinal hernia repair of exclusively male patients with primary unilateral inguinal hernia, it was also demonstrated that, as in the literature [1–6], young age (≤ 40 years) and preoperative pain are important influencing factors for onset of chronic pain. The present multivariable analysis has revealed that pain at rest, pain on exertion, and chronic pain requiring treatment was highly affected by preoperative pain and young age. But that was also true for small hernia defects. For a small hernia defect the risk of pain at rest, on exertion, and requiring treatment appeared to be highly significantly greater than for a large hernia defect. One explanation for this could be that a patient who is willing to undergo surgery for even a smaller inguinal hernia is more sensitive to pain [1], and already experiences preoperative pain. But the issue of the indication for surgery must also be addressed. Was the inguinal pain really related to a small inguinal hernia or was this due to other causes that also persisted after inguinal hernia repair? Other causes of inguinal pain must be effectively ruled out.

This clearly demonstrates that young patients with a small inguinal hernia (EHS I: < 1.5 cm) and inguinal pain are at highest risk for onset of chronic pain following laparoscopic inguinal hernia repair. Accordingly, a well-founded indication for surgery is of strictly crucial importance for these patients. The patient should definitely be made aware of this before signing the declaration of informed consent form for surgery. If the indication is correct, the operation should be performed in accordance with the evidence-based guidelines for the TAPP technique [9, 10].

Likewise, patients with higher BMI value (≥ 25.0) had a highly significant influence on the risk of pain at rest, on exertion, and chronic pain requiring treatment after TAPP operation. Overweight or obesity, in particular in male patients makes additional demands on the surgeon during conduct of TAPP. Therefore commensurate caution must be exercised when performing surgery for overweight patients.

In addition to the most important influencing factors for onset of chronic pain after laparoscopic inguinal hernia surgery (young age, preoperative pain, small hernia defect, and higher BMI), there are other factors affecting onset of chronic pain. These include postoperative complications and the use of penetrating tackers for mesh fixation. As demonstrated in five meta-analyses, the use of penetrating mesh fixation compared with glue fixation led to significantly more chronic inguinal pain [17–21], but the evidence is mostly of low or moderate quality. Unlike the aforementioned meta-analyses, on comparing tack mesh fixation vs. non-fixation Sajid et al. [15] did not find any difference in the chronic pain rates. Likewise, a registry-based Danish study did not find any difference in chronic pain rates after TAPP operation on comparing mesh fixation with fibrin glue vs. tacks [24]. In our study, the influence of penetrating tacks on chronic pain was only confirmed for pain on exertion on comparing non-fixation vs. tack fixation. In their systematic review, Lederhuber et al. [22] concluded that there is still a lack of high-quality evidence for differences between the assessed mesh fixation techniques. Therefore, more high-quality multicenter studies are needed [22]. The findings of our study at least suggest that other factors, such as a small hernia, preoperative pain, younger age, and higher BMI, have a greater impact on the development of chronic pain than does the fixation technique.

Likewise, postoperative complications can trigger inguinal pain. Therefore, an appropriate response must be taken to any development of postoperative complications after TAPP operation to prevent the onset of chronic inguinal pain.

The potential weakness of this study is its non-randomization and the voluntary participation in the internet-based registration. These could lead to selection bias, which can be balanced by the large case number of the study. Furthermore, the registry does not contain any data on how the peritoneum was closed.

In summary, it can be stated that there are several influencing factors for pain at rest, on exertion, and chronic pain requiring treatment following primary unilateral inguinal hernia repair in male patients in TAPP technique. Younger patient age, preoperative pain, smaller hernia defect size, and higher BMI value have a highly significant influence. Other potentially influencing factors are penetrating mesh fixation and development of postoperative complications. Through a well-founded indication, and observance of the technical guidelines for evidence-based conduct of TAPP, it may be possible to prevent chronic pain after TAPP operation.

## Appendix

### Herniamed Study Group

#### Scientific board

Köckerling, Ferdinand (Chairman) (Berlin); Bittner, Reinhard (Rottenburg); Fortelny, René (Wien); Jacob, Dietmar (Berlin); Koch, Andreas (Cottbus); Kraft, Barbara (Stuttgart); Kuthe, Andreas (Hannover); Lammers, Bernhard (Neuss); Lippert, Hans (Magdeburg): Lorenz, Ralph (Berlin); Mayer, Franz (Salzburg); Niebuhr, Henning (Hamburg); Peiper, Christian (Hamm); Pross, Matthias (Berlin); Reinpold, Wolfgang (Hamburg); Simon, Thomas (Weinheim); Stechemesser, Bernd (Köln); Unger, Solveig (Chemnitz); Weyhe, Dirk (Oldenburg); Zarras, Konstantinos (Düsseldorf).

#### Participants

Ahmetov, Azat (Saint-Petersburg); Alapatt, Terence Francis (Frankfurt/Main); Albayrak, Nurretin (Herne); Amann, Stefan (Neuendettelsau); Anders, Stefan (Berlin); Anderson, Jürina (Würzburg); Antoine, Dirk (Leverkusen); Apfelstedt, Heinrich (Solingen); Arndt, Anatoli (Elmshorn); Aschenbrenner, Michael (Spittal/Drau); Asperger, Walter (Halle); Avram, Iulian (Saarbrücken); Baikoglu-Endres, Corc (Weißenburg i. Bay.); Bandowsky, Boris (Damme); Barkus; Jörg (Velbert); Becker, Matthias (Freital); Behrend, Matthias (Deggendorf); Berkhoff, Christian (Fulda); Beuleke, Andrea (Burgwedel); Birk, Dieter (Bietigheim-Bissingen); Bittner, Reinhard (Rottenburg); Blaha, Pavel (Zwiesel); Blumberg, Claus (Lübeck); Böckmann, Ulrich (Papenburg); Böhle, Arnd Steffen (Bremen); Bolle, Ludger (Berlin); Borchert, Erika (Grevenbroich); Born, Henry (Leipzig); Brabender, Jan (Köln); Breitenbuch von, Philipp (Radebeul); Brož, Miroslav (Ebersbach); Brückner, Torsten (Gießen); Brütting, Alfred (Erlangen); Buchert, Annette (Mallersdorf-Pfaffenberg); Buchholz, Torsten (Aurich); Budzier, Eckhard (Meldorf); Burchett, Bert (Teterow); Burghardt, Jens (Rüdersdorf); Cejnar, Stephan-Alexander (München); Chirikov, Ruslan (Dorsten); Claußnitzer, Christian (Ulm); Comman, Andreas (Bogen); Crescenti, Fabio (Verden/Aller); Daniels, Thies (Hamburg); Dapunt, Emanuela (Bruneck); Decker, Georg (Berlin); Demmel, Michael (Arnsberg); Descloux, Alexandre (Baden); Deusch, Klaus-Peter (Wiesbaden); Dick, Marcus (Neumünster); Dieterich, Klaus (Ditzingen); Dietz, Harald (Landshut); Dittmann, Michael (Northeim); Drummer, Bernhard (Forchheim); Eckermann, Oliver (Luckenwalde); Eckhoff (Jörn /Hamburg); Ehmann, Frank (Grünstadt); Eisenkrein, Alexander (Düren); Elger, Karlheinz (Germersheim); Engelhardt, Thomas (Erfurt); Erichsen, Axel (Friedrichshafen); Eucker, Dietmar (Bruderholz); Fackeldey, Volker (Kitzingen); Faddah, Yousif (Kamenz); Farke, Stefan (Delmenhorst); Faust, Hendrik (Emden); Federmann, Georg (Seehausen); Fiedler, Michael (Eisenberg); Fikatas, Panagiotis (Berlin); Firl, Michaela (Perleberg); Fischer, Ines (Wiener Neustadt); Fleischer, Sabine (Dinslaken); Fortelny, René H. (Wien); Franczak, Andreas (Wien); Franke, Claus (Düsseldorf); Frankenberg von, Moritz (Salem); Frehner, Wolfgang (Ottobeuren); Friedhoff, Klaus (Andernach); Friedrich, Jürgen (Essen); Frings, Wolfram (Bonn); Fritsche, Ralf (Darmstadt); Frommhold, Klaus (Coesfeld); Frunder, Albrecht (Tübingen); Fuhrer, Günther (Reutlingen); Garlipp, Ulrich (Bitterfeld-Wolfen); Gassler, Harald (Villach); Gawad, Karim A. (Frankfurt/Main); Gehrig, Tobias (Sinsheim); Gerdes, Martin (Ostercappeln); Germanov, German (Halberstadt); Gilg, Kai-Uwe (Hartmannsdorf); Glaubitz, Martin (Neumünster); Glauner-Goldschmidt, Kerstin (Werne); Glutig, Holger (Meissen); Gmeiner, Dietmar (Bad Dürrnberg); Göring, Herbert (München); Grebe, Werner (Rheda-Wiedenbrück); Grothe, Dirk (Melle); Günther, Thomas (Dresden); Gürtler, Thomas (Zürich); Hache, Helmer (Löbau); Hämmerle, Alexander (Bad Pyrmont); Haffner, Eugen (Hamm); Hain, Hans-Jürgen (Gross-Umstadt); Halter, Christian Jörn (Recklinghausen); Hammans, Sebastian (Lingen); Hampe, Carsten (Garbsen); Hanke, Stefan (Halle); Harrer, Petra (Starnberg); Hartung, Peter (Werne); Heinzmann, Bernd (Magdeburg); Heise, Joachim Wilfried (Stolberg); Heitland, Tim (München); Helbling, Christian (Uznach/Schweiz); Hellinger, Achim (Fulda); Hempen, Hans-Günther (Cloppenburg); Henneking, Klaus-Wilhelm (Bayreuth); Hennes, Norbert (Duisburg); Herdter, Christian (Gelsenkirchen); Hermes, Wolfgang (Weyhe); Herzing, Holger (Höchstadt); Hessler, Christian (Bingen); Heuer, Matthias (Herten); Hildebrand, Christiaan (Langenfeld); Höferlin, Andreas (Mainz); Hoffmann, Henry (Basel); Hoffmann, Michael (Kassel); Hofmann, Eva M. (Frankfurt/Main); Horbach, Thomas (Fürth); Hornung, Frederic (Wolfratshausen); Hudak, Attila (Suhl); Hübel-Abe, Jan (Ilmenau); Hügel, Omar (Hannover); Hüttemann, Martin (Oberhausen); Hüttenhain, Thomas (Mosbach); Hunkeler, Rolf (Zürich); Imdahl, Andreas (Heidenheim); Iseke, Udo (Duderstadt); Isemer, Friedrich-Eckart (Wiesbaden); Jablonski, Herbert Gustav (Sögel); Jacob, Dietmar (Berlin); Jansen-Winkeln, Boris (Leipzig); Jantschulev, Methodi (Waren); Jenert, Burghard (Lichtenstein); Jugenheimer, Michael (Herrenberg); Junge, Karsten (Aachen); Kaaden, Stephan (Neustadt am Rübenberge); Käs, Stephan (Weiden); Kahraman, Orhan (Hamburg); Kaiser, Christian (Westerstede); Kaiser, Gernot Maximilian (Kamp-Lintfort); Kaiser, Stefan (Kleinmachnow); Karch, Matthias (Eichstätt); Kasparek, Michael S. (München); Kastl, Sigrid (Braunau am Inn); Keck, Heinrich (Wolfenbüttel); Keller, Hans W. (Bonn); Kewer, Jans Ludolf (Tuttlingen); Kienzle, Ulrich (Karlsruhe); Kipfmüller, Brigitte (Köthen); Kirsch, Ulrike (Oranienburg); Klammer, Frank (Ahlen); Klatt, Richard (Hagen); Klein, Karl-Hermann (Burbach); Kleist, Sven (Berlin); Klobusicky, Pavol (Bad Kissingen); Kneifel, Thomas (Datteln); Knolle, Winfried (Pritzwalk); Knoop, Michael (Frankfurt/Oder); Knotter, Bianca (Mannheim); Koch, Andreas (Cottbus); Koch, Andreas (Münster); Köckerling, Ferdinand (Berlin); Köhler, Gernot (Linz); König, Oliver (Buchholz); Kornblum, Hans (Tübingen); Krämer, Dirk (Bad Zwischenahn); Kraft, Barbara (Stuttgart); Kratsch, Barthel (Dierdorf/Selters); Krausbeck, Matthias (Schwerin); Kreissl, Peter (Ebersberg); Krones, Carsten Johannes (Aachen); Kronhardt, Heinrich (Neustadt am Rübenberge); Kruse, Christinan (Aschaffenburg); Kube, Rainer (Cottbus); Kühlberg, Thomas (Berlin); Kühn, Gert (Freiberg); Kuhn, Roger (Gifhorn); Kusch, Eduard (Gütersloh); Kuthe, Andreas (Hannover); Ladberg, Ralf (Bremen); Ladra, Jürgen (Düren); Lahr-Eigen, Rolf (Potsdam); Lainka, Martin (Wattenscheid); Lalla, Thomas (Oschersleben); Lammers, Bernhard J. (Neuss); Lancee, Steffen (Alsfeld); Lange, Claas (Berlin); Langer, Claus (Göttingen); Laps, Rainer (Ehringshausen); Larusson, Hannes Jon (Pinneberg); Lauschke, Holger (Duisburg); Lechner-Puschnig, Marina (Klagenfurt am Wörthersee/Österreich); Leher, Markus (Schärding); Leidl, Stefan (Waidhofen/Ybbs); Leisten, Edith (Köln); Lenz, Stefan (Berlin); Liedke, Marc Olaf (Heide); Lienert, Mark (Duisburg); Limberger, Andreas (Schrobenhausen); Limmer, Stefan (Würzburg); Locher, Martin (Kiel); Loghmanieh, Siawasch (Viersen); Lorenz, Ralph (Berlin); Luedtke, Clinton (Kusel); Luther, Stefan (Wipperfürth); Luyken, Walter (Sulzbach-Rosenberg); Mallmann, Bernhard (Krefeld); Manger, Regina (Schwabmünchen); Maurer, Stephan (Münster); May, Jens Peter (Schönebeck); Mayer, Franz (Salzburg); Mayer, Jens (Schwäbisch Gmünd); Mellert, Joachim (Höxter); Menzel, Ingo (Weimar); Meurer, Kirsten (Bochum); Meyer, Moritz (Ahaus); Mirow, Lutz (Zwickau); Mittag-Bonsch, Martina (Crailsheim); Möbius, Ekkehard (Braunschweig); Mörder-Köttgen, Anja (Freiburg); Moesta, Kurt Thomas (Hannover); Mugomba, Gilbert (Dannenberg); Moldenhauer, Ingolf (Braunschweig); Morkramer, Rolf (Radevormwald); Mosa, Tawfik (Merseburg); Müller, Hannes (Schlanders); Müller, Volker (Nürnberg); Münzberg, Gregor (Berlin); Murr, Alfons (Vilshofen); Mussack, Thomas (St. Gallen); Nartschik, Peter (Quedlinburg); Nasifoglu, Bernd (Ehingen); Neumann, Jürgen (Haan); Neumeuer, Kai (Paderborn); Niebuhr, Henning (Hamburg); Nix, Carsten (Walsrode); Nölling, Anke (Burbach); Nostitz, Friedrich Zoltán (Mühlhausen); Obermaier (Straubing); Öz-Schmidt, Meryem (Hanau); Oldorf, Peter (Usingen); Olivieri, Manuel (Pforzheim); Passon, Marius (Freudenberg); Pawelzik, Marek (Hamburg); Pein, Tobias (Hameln); Peiper, Christian (Hamm); Peiper, Matthias (Essen); Pertl, Alexander (Spittal/Drau); Philipp, Mark (Rostock); Pickart, Lutz (Bad Langensalza); Pizzera, Christian (Graz); Pöllath, Martin (Sulzbach-Rosenberg); Pöschmann, Enrico (Thalwil); Possin, Ulrich (Laatzen); Prenzel, Klaus (Bad Neuenahr-Ahrweiler); Pröve, Florian (Goslar); Pronnet, Thomas (Fürstenfeldbruck); Pross, Matthias (Berlin); Puff, Johannes (Dinkelsbühl); Rabl, Anton (Passau); Raggi, Matthias Claudius (Stuttgart); Rapp, Martin (Neunkirchen); Reck, Thomas (Püttlingen); Reinpold, Wolfgang (Hamburg); Renter, Marc Alexander (Moers); Reuter, Christoph (Quakenbrück); Radke, Alexander (Thun/Zweisimmen); Richter, Jörg (Winnenden); Riemann, Kerstin (Alzenau-Wasserlos); Riesener, Klaus-Peter (Marl); Rodehorst, Anette (Otterndorf); Roehr, Thomas (Rödental); Rössler, Michael (Rüdesheim am Rhein); Roncossek (Bremerhaven); Rosniatowski, Rolland (Marburg); Roth Hartmut (Nürnberg); Sardoschau, Nihad (Saarbrücken); Sauer, Gottfried (Rüsselsheim); Sauer, Jörg (Arnsberg); Seekamp, Axel (Freiburg); Seelig, Matthias (Bad Soden); Seidel, Hanka (Eschweiler); Seiler, Christoph Michael (Warendorf); Seltmann, Cornelia (Hachenburg); Senkal, Metin (Witten); Shamiyeh, Andreas (Linz); Shang, Edward (München); Siemssen, Björn (Berlin); Sievers, Dörte (Hamburg); Silbernik, Daniel (Bonn); Simon, Thomas (Weinheim); Sinn, Daniel (Olpe); Sinner, Guy (Merzig); Sinning, Frank (Nürnberg); Smaxwil, Constatin Aurel (Stuttgart); Sörensen, Björn (Lauf an der Pegnitz): Sucke, Jochen Markus (Gießen); Syga, Günter (Bayreuth); Schabel, Volker (Kirchheim/Teck); Schadd, Peter (Euskirchen); Schassen von, Christian (Hamburg); Schattenhofer, Thomas (Vilshofen); Scheibel, Mike (Krefeld); Schelp, Lothar (Wuppertal); Scherf, Alexander (Pforzheim); Scheuerlein, Hubert (Paderborn); Scheyer, Mathias (Bludenz); Schilling, André (Kamen); Schimmelpenning, Hendrik (Neustadt in Holstein); Schinkel, Svenja (Kempten); Schmid, Michael (Gera); Schmid, Thomas (Innsbruck); Schmidt, Ulf (Mechernich); Schmitz, Heiner (Jena); Schmitz, Ronald (Altenburg); Schöche, Jan (Borna); Schoenen, Detlef (Schwandorf); Schrittwieser, Rudolf (Bruck an der Mur); Schroll, Andreas (München); Schubert, Daniel (Saarbrücken); Schüder, Gerhard (Wertheim); Schürmann, Rainer (Steinfurt); Schultz, Christian (Bremen-Lesum); Schultz, Harald (Landstuhl); Schulze, Frank P. (Mülheim an der Ruhr); Schulze, Thomas (Dessau-Roßlau); Schumacher, Franz-Josef (Oberhausen); Schwab, Robert (Koblenz); Schwandner, Thilo (Lich); Schwarz, Jochen Günter (Rottenburg); Schymatzek, Ulrich (Eitorf); Spangenberger, Wolfgang (Bergisch-Gladbach); Sperling, Peter (Montabaur); Staade, Katja (Düsseldorf); Staib, Ludger (Esslingen); Staikov, Plamen (Frankfurt am Main); Stamm, Ingrid (Heppenheim); Stark, Wolfgang (Roth); Stechemesser, Bernd (Köln); Steinhilper, Uz (München); Stengl, Wolfgang (Nürnberg); Stern, Oliver (Hamburg); Stöltzing, Oliver (Meißen); Stolte, Thomas (Mannheim); Stopinski, Jürgen (Schwalmstadt); Stratmann, Gerald (Goch); Straßburger, Harald (Alfeld); Stubbe, Hendrik (Güstrow/); Stülzebach, Carsten (Friedrichroda); Tepel, Jürgen (Osnabrück); Terzić, Alexander (Wildeshausen); Teske, Ulrich (Essen); Thasler, Wolfgang (München); Tichomirow, Alexej (Brühl); Tillenburg, Wolfgang (Marktheidenfeld); Timmermann, Wolfgang (Hagen); Tomov, Tsvetomir (Koblenz); Train, Stefan H. (Gronau); Trauzettel, Uwe (Plettenberg); Triechelt, Uwe (Langenhagen); Ulbricht, Wolfgang (Breitenbrunn); Ulcar, Heimo (Schwarzach im Pongau); Ungeheuer, Andreas (München); Unger, Solveig (Chemnitz); Utech, Markus (Gelsenkirchen); Verweel, Rainer (Hürth); Vogel, Ulrike (Berlin); Voigt, Rigo (Altenburg); Voit, Gerhard (Fürth); Volkers, Hans-Uwe (Norden); Volmer, Ulla (Berlin); Vossough, Alexander (Neuss); Wallasch, Andreas (Menden); Wallner, Axel (Lüdinghausen); Warscher, Manfred (Lienz); Warwas, Markus (Bonn); Weber, Jörg (Köln); Weber, Uwe (Eggenfelden); Weihrauch, Thomas (Ilmenau); Weiß, Heiko (Aue); Weiß, Johannes (Schwetzingen); Weißenbach, Peter (Neunkirchen); Werner, Uwe (Lübbecke-Rahden); Wessel, Ina (Duisburg); Weyhe, Dirk (Oldenburg); Wicht, Sebastian (Bützow); Wieber, Isabell (Köln); Wiens, Matthias (Affoltern); Wiesmann, Aloys (Rheine); Wiesner, Ingo (Halle); Withöft, Detlef (Neutraubling); Woehe, Fritz (Sangerhausen); Wolf, Claudio (Neuwied); Wolkersdörfer, Toralf (Pößneck); Yaksan, Arif (Wermelskirchen); Yildirim, Can (Lilienthal); Yildirim, Selcuk (Berlin); Zarras, Konstantinos (Düsseldorf); Zeller, Johannes (Waldshut-Tiengen); Zhorzel, Sven (Agatharied); Zuz, Gerhard (Leipzig).
